# Variant Amino Acid Residues Alter the Enzyme Activity of Peanut Type 2 Diacylglycerol Acyltransferases

**DOI:** 10.3389/fpls.2017.01751

**Published:** 2017-10-16

**Authors:** Ling Zheng, Jay Shockey, Fei Bian, Gao Chen, Lei Shan, Xinguo Li, Shubo Wan, Zhenying Peng

**Affiliations:** ^1^College of Life Science, Shandong University, Jinan, China; ^2^Shandong Provincial Key Laboratory of Genetic Improvement, Ecology and Physiology of Crops, Department of Bio-Tech Research Center, Shandong Academy of Agricultural Sciences, Jinan, China; ^3^United States Department of Agriculture, Southern Regional Research Center, Agricultural Research Service, New Orleans, LA, United States

**Keywords:** diacylglycerol acyltransferase, triacylglycerol, lipid, transformation, peanut oil

## Abstract

Diacylglycerol acyltransferase (DGAT) catalyzes the final step in triacylglycerol (TAG) biosynthesis via the acyl-CoA-dependent acylation of diacylglycerol. This reaction is a major control point in the Kennedy pathway for biosynthesis of TAG, which is the most important form of stored metabolic energy in most oil-producing plants. In this study, Arachis hypogaea type 2 DGAT (AhDGAT2) genes were cloned from the peanut cultivar ‘Luhua 14.’ Sequence analysis of 11 different peanut cultivars revealed a gene family of 8 peanut DGAT2 genes (designated AhDGAT2a-h). Sequence alignments revealed 21 nucleotide differences between the eight ORFs, but only six differences result in changes to the predicted amino acid (AA) sequences. A representative full-length cDNA clone (AhDGAT2a) was characterized in detail. The biochemical effects of altering the AhDGAT2a sequence to include single variable AA residues were tested by mutagenesis and functional complementation assays in transgenic yeast systems. All six mutant variants retained enzyme activity and produced lipid droplets in vivo. The N6D and A26P mutants also displayed increased enzyme activity and/or total cellular fatty acid (FA) content. N6D mutant mainly increased the content of palmitoleic acid, and A26P mutant mainly increased the content of palmitic acid. The A26P mutant grew well both in the presence of oleic and C18:2, but the other mutants grew better in the presence of C18:2. AhDGAT2 is expressed in all peanut organs analyzed, with high transcript levels in leaves and flowers. These levels are comparable to that found in immature seeds, where DGAT2 expression is most abundant in other plants. Over-expression of AhDGAT2a in tobacco substantially increased the FA content of transformed tobacco seeds. Expression of AhDGAT2a also altered transcription levels of endogenous tobacco lipid metabolic genes in transgenic tobacco, apparently creating a larger carbon ‘sink’ that supports increased FA levels.

## Introduction

Peanuts are used in a variety of important food products, including whole roasted peanuts, peanut butter, and as an essential ingredient of various entrees in cuisines from around the world. Peanut oil is also a valuable commodity used in cooking. It possesses a mild flavor, and its high oleic acid/linoleic acid ratio (O/L ratio) conveys a high smoke point and low rancidity relative to many other cooking oils. Peanut oil mostly consists of triacylglycerols (TAGs), the primary form of stored carbon in peanut plants. DGAT catalyzes the final reaction of the Kennedy pathway (Kennedy, 1961) of TAG biosynthesis, and due to its influence over both the quantitative and qualitative aspects of TAG profiles, this enzyme is considered a ‘gatekeeper’ of seed lipid metabolism (Weselake et al., 2009). Many laboratories have focused on DGATs because of their important roles in TAG synthesis. Studies of both type-1 (*DGAT1*) and type-2 DGAT (*DGAT2*) genes have provided many useful insights into the properties of these enzymes, and have shown that *DGAT* over-expression often greatly increases the oil content of transgenic organisms (Cao and Huang, 1986; Cases et al., 1998; Hobbs et al., 1999; Bouvier-Navé et al., 2000; Jako et al., 2001; Lardizabal et al., 2001; He et al., 2004; Kroon et al., 2006; Saha et al., 2006; Shockey et al., 2006; Wang et al., 2006; Burgal et al., 2008; Lardizabal et al., 2008; Zheng et al., 2008; Andrianov et al., 2010; Durrett et al., 2010; Cao, 2011; Cao et al., 2013; Gong et al., 2013; Guo et al., 2013; Misra et al., 2013; Niu et al., 2013; Peng et al., 2013; Xu et al., 2013; Zhang et al., 2013; Aymé et al., 2014; Chi et al., 2014; Dey et al., 2014).

DGAT1 and DGAT2 are responsible for the bulk of TAG synthesis, and seem to play at least partially non-redundant roles in TAG synthesis in plants and other organisms (Shockey et al., 2006). In Arabidopsis and soybeans, DGAT1 appears to play the major role in seed oil accumulation (Hobbs et al., 1999; Jako et al., 2001; Zheng et al., 2008). In castor bean and tung tree, DGAT2 appears to drive accumulation of unusual fatty acids (FAs) in their respective seed oils (Kroon et al., 2006; Burgal et al., 2008), although castor DGAT1 likely contributes to the near-purity of ricinoleic acid (a hydroxylated FA) in castor oil as well (He et al., 2004). In contrast, burning bush (*Euonymus alatus*, whose seed oil contains high levels of TAGs containing *sn*-3 acetyl groups, rather than long-chain FAs) DGAT2 has only long-chain acyltransferase activity and not acetyltransferase activity, whereas *E. alatus* DGAT1 possesses both long-chain acyl- and acetyltransferase activity *in vitro* (Durrett et al., 2010). Dey et al. (2014) identified two structurally novel DGAT2 isozymes from the yeast *Candida tropicalis* SY005; CtDGAT2a and CtDGAT2b differ by 9 amino acid (AA). Comparison of the predicted 2D structures of CtDGAT2a and CtDGAT2b revealed differences at several positions in alpha-helices and beta-sheets. Through analysis of the predicted 3D structures of CtDGAT2a and CtDGAT2b, the authors found two additional large side chain loops in CtDGAT2a, whereas only one such loop is formed in CtDGAT2b. Though the differences in the predicted primary, 2D, and 3D protein structures were significant, CtDGAT2a and CtDGAT2b are both functionally active, and interestingly, CtDGAT2b more efficiently produced storage lipids in a heterologous yeast system than did CtDGAT2a (Dey et al., 2014).

Given its role in controlling the FA composition of the final storage lipid pool, many studies have sought to determine the acyl-CoA and/or diacylglycerol (DAG) preferences of various plant DGAT enzymes (Lung and Weselake, 2006). When *Jatropha curcas* DGAT1 (JcDGAT1) was over-expressed in transgenic *Arabidopsis thaliana*, the levels of saturated FAs in seeds did not change, whereas the levels of oleic acid (C18:1) decreased and linolenic acid (C18:3) increased (Misra et al., 2013). Yeast expression of *Thalassiosira pseudonana* DGAT2 leads to increased C18:1 and a concomitant decrease of palmitic acid (C16:0), relative to Arabidopsis DGAT1 (Xu et al., 2013). A DGAT2 from *Claviceps purpurea* prefers ricinoleic acid as an acyl donor over linoleic acid (C18:2), C18:1, or C18:3 (Mavraganis et al., 2010). Co-expression of the *Stokesia laevis* epoxygenase with either type-1 or type-2 *Vernonia galamensis* DGATs in petunia leaves and soybean somatic embryos led to efficient production of epoxygenated FAs (vernolic acid: ∼15 and 26%, respectively, for DGAT1 and DGAT2 in soybean embryos) (Li et al., 2010).

One of the aims of this work is to search for key AA residues that influence the catalytic properties of the DGAT reaction. Some AA substitutions/insertions in bovine or plant DGAT1s significantly change the enzymatic activities resulting in changes in milk fat or plant seed oil content, respectively. A non-conservative substitution of lysine by alanine in bovine DGAT1 lowers milk fat content and alters other milk characteristics (Grisart et al., 2002; Winter et al., 2002). A phenylalanine insertion at position 469 in maize DGAT1-2 is responsible for increased oil and C18:1 contents, and ectopic expression of the high-oil *DGAT1-2* allele increases oil and C18:1 content by up to 41 and 107%, respectively (Zheng et al., 2008). The substrate preferences of Arabidopsis DGAT2 had long been mysterious, due to inabilities to successfully overexpress this enzyme in transgenic systems (Lardizabal et al., 2001; Burgal et al., 2008); recent experiments with a codon-optimized version of the *AtDGAT2* gene restored TAG accumulation in the storage lipid-deficient *Saccharomyces cerevisiae* mutant strain H1246, and showed that AtDGAT2 preferentially incorporated palmitoleic acid, C18:2 and C18:3 (Zhou et al., 2013; Aymé et al., 2014). Stone et al. (2006) mutated a highly conserved, but previously uncharacterized HPHG sequence in murine DGAT2 and found that it is required for full enzymatic function. But to date, little is known about the effects of specific AA substitutions on DGAT enzymatic activity and substrate specificity, including those of plant DGAT2s.

In this study, we identified eight closely related *AhDGAT2* genes, which we have called *AhDGAT2a-h*. In order to attempt to identify AA changes that can affect DGAT2 activity, we chose to analyze DGAT2 sequences from a collection of peanut varieties and to compare the relative activities of AA-substituted variants by expression in yeast. The effects of plant seed overexpression of a representative peanut *DGAT2* gene are also described.

## Materials and Methods

### Plant and Yeast Materials

Eleven peanut cultivars were investigated (Supplementary Table S1). Functional characterization of *AhDGAT2* genes was carried out after transformation of tobacco (*Nicotiana tabacum*) cultivar ‘SR1’ and TAG- and sterol lipid-deficient bakers’ yeast (*S. cerevisiae*) strain H1246 (Sandager et al., 2002).

### Cloning of Full-Length *AhDGAT2* cDNA

Total RNA was isolated from peanut pods 25 days after flowering (DAF). Five microgram of RNA was reverse transcribed (in a 20 μl reaction volume) into first-strand cDNAs using a cDNA synthesis kit (Invitrogen, United States). By analyzing the conserved domains of the *Medicago truncatula* and castor DGAT nucleotide sequences, we designed a pair of primers (AhD2-S and AhD2-A, Supplementary Table S2) that successfully amplified a 197 bp fragment of the central portion of the coding sequence. Twenty microliter PCR mixtures, containing 1 μl cDNA, 1 μl of each primer (10 μM), 2 μl 10 × PCR buffer, 2 μl dNTPs (2.5 mM each), and 1 unit of *Pyrococcus furiosus* (*Pfu*) DNA polymerase (Invitrogen, United States) were employed. Reactions were denatured at 94°C for 5 min, followed by 30 cycles of 30 s at 94°C, 30 s at 50°C, 30 s at 72°C, then 10 min at 72°C. PCRs were performed using a PCR Thermal Cycler Dice-TP600 (Takara, Japan). The *AhDGAT2* product was purified using a MinElute^TM^ Gel Extraction Kit (Qiagen, Germany), cloned into a pMD18-T vector (Takara, Japan), then sequenced.

A full-length peanut *DGAT2* cDNA from the ‘Luhua 14’ cultivar was obtained using the SMART^TM^ RACE cDNA Amplification Kit (Clontech, United States). One microgram of total RNA extracted from pods at 25 DAF was used to synthesize the cDNA following the manufacturer’s protocol. RACE primers (AhD2-3O and AhD2-3I, AhD2-5O and AhD2-5I, Supplementary Table S2) were based on the sequence of the peanut *DGAT2* fragment described above. PCR reactions were performed according to the manufacturer’s protocol. The sequences from cloned fragments were assembled into a predicted full-length ORF.

Based on the assembled RACE sequences, the full-length peanut *DGAT2* ORF was amplified using gene-specific primers (AhD2-FS and AhD2-FA, Supplementary Table S2). The PCR mixtures (in 20 μl volumes), contained 1 μl cDNA, 1 μl of each primer (10 μM), 2 μl PCR buffer (10 × buffer), 4 μl dNTPs (2.5 mM each), and 1 unit of *Pfu* DNA polymerase. The reaction was denatured at 94°C for 5 min, followed by 30 cycles of 30 s at 94°C, 30 s at 60°C, 1 min 20 s at 72°C, then 10 min at 72°C. The full-length peanut *DGAT2* amplicon was purified from an agarose gel and cloned into a pMD18-T vector for sequencing.

### Sequence Alignments and Phylogenetic Comparisons of Different Peanut *DGAT2* Sequences

The ORFs of different *DGAT2* cDNAs from 10 selected peanut cultivars were amplified, sequenced, and compared. Nucleotide sequence translations, AA sequence alignments, and phylogenetic tree analyses were conducted using DNAMAN software (Lynnon Biosoft, United States). Database searches were conducted using the BLAST program at the National Center for Biotechnology Information (NCBI) database (Altschul et al., 1990). Protein motifs were identified using the Conserved Domain Search Service program at the NCBI database (Marchler-Bauer et al., 2011).

### RNA Isolation and Q-PCR

Total RNA was isolated from seven different seed developmental stages according to DAF stages (7–14 DAF; 15–22 DAF; 23–30 DAF; 31–37 DAF; 38–45 DAF; 46–53 DAF; 54–61 DAF; 62–69 DAF), using the CTAB method (Gambino et al., 2008). Root, leaf, stem, and flower samples from wild-type (WT) peanuts were treated in the same manner. First-strand cDNA was synthesized from the target mRNA using a cDNA synthesis kit. The primers (AhD2-SF and AhD2-SR, Supplementary Table S2), for specific amplification of *AhDGAT2* cDNA, were designed to amplify a 176 bp portion of the target gene. Primers AhActin-S and AhActin-A (Supplementary Table S2) that amplify a 112 bp fragment of this gene were used as a control in these experiments. Q-PCR was conducted in triplicate using SYBR Green Mix under reaction conditions: 2 min at 50°C, 10 min at 95°C followed by 40 cycles of 15 s at 95°C, 1 min at 60°C, and 15 s at 95°C followed by ABI7500 Fast Real-Time PCR System machine.

### Construction of Different Mutated Version of *AhDGAT2a* Genes

**FIGURE 1:**
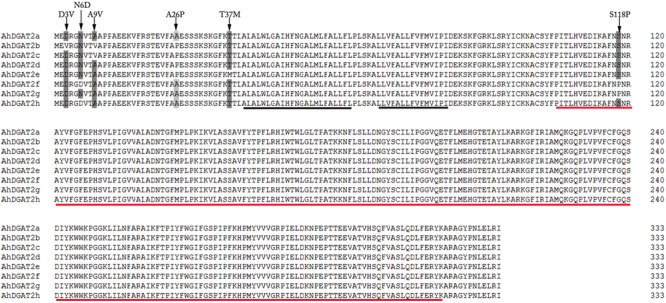
Amino acid sequence comparisons of AhDGAT2a-h. The black lines highlight the two predicted transmembrane domains. The red line shows the conserved LPLAT_MGAT-like domain (amino acids 104–321). D3V, N6D, A9V, A26P, T37M, and S118P indicate the six variant amino acid residues, where the first letter represents the consensus residue, followed by the numerical location of the residue, and the letter representing the alternate residue present in the protein variant.

In order to test the influence of each AA difference on the biochemical properties of the parental enzyme, we created mutated copies of the *AhDGAT2a* gene, either by site-specific mutagenesis using specific forward primers (D3V and A9V, Supplementary Table S2) paired with the parental reverse primer DGAT2AR, or by direct gene synthesis (T37M) (synthesized by Sangon, China). The template sequences of N6D, A26P, and S118P were *AhDGAT2h, AhDGAT2d*, and *AhDGAT2g*, respectively. *AhDGAT2a* is regarded as the standard, ‘parental’ sequence. The locations of mutant AAs of the sequences are shown in **Figure 1**. The six variant residues were named D3V, N6D, A9V, A26P, T37M, and S118P, respectively.

### Yeast Transformation and Plate Growth Assays

The ORFs of *AhDGAT2a* and the six mutant sequences were amplified by PCR, then cut with *Not*I and *Pac*I, and cloned into the same sites in multiple cloning site 1 (MCS1) of the galactose-inducible yeast expression vector pESC-URA (Agilent Technologies, United States), as *N*-terminal His_6_ fusions. The plasmids were transformed into competent cells of *S. cerevisiae* strain H1246, and transgenic colonies were selected on SD-URA media. Pooled colonies from each of the strains listed below were grown in selective liquid media, then back-diluted into liquid media containing galactose or raffinose to induce protein expression. Serial dilutions of each culture were spotted on solid galactose media lacking uracil, and containing either no FAs, or 1–3 mM oleate or linoleate. These cultures were grown at 30°C and the growth status photographed by a Nikon camera D7000 at different time points. Strains bearing empty pESC-URA plasmid or plasmid which contains tung DGAT1 (Shockey et al., 2006) were used as negative and positive controls, respectively.

### Nile Red Staining and Fluorescence Analysis

Aliquots of stationary phase cells (400 μl) were pelleted and washed twice in PBS, gently dispersed in 20 μl of PBS, then mixed with 5 μl Nile Red (1 μg/μl) (Sandager et al., 2002; Siloto et al., 2009). The stained cells were incubated in the dark for 10 min at 30°C, then washed twice in PBS and diluted into 100 μl of PBS. The stained cells were observed and photographed with a fluorescence microscope (Olympus IX71-A12FL/PH, Japan) containing a digital camera. We used Image-Pro plus software (Media Cybernetics, Rockville, MD, United States) to analyze the fluorescence intensity of transgenic yeasts.

### Western Blotting

Yeast protein was extracted from intact cells using Yeast Total Protein Extraction kit (Sangon Biotech, China); protein concentrations were determined by the Bradford method (Bradford, 1976). Fifty microgram samples were separated by SDS-PAGE and used for western blot analysis. Western blots were performed as previously described (Cao et al., 1999). The primary antisera were anti-His (Proteintech, United States) and anti-Actin (Proteintech, United States) diluted 1:2,000. The secondary antibody was goat anti-mouse IgG-alkaline phosphatase conjugate (Proteintech, United States) diluted 1:3,000.

### Genetic Transformation of WT Tobacco

Specific primers were designed to introduce a *Bam*HI restriction site upstream of the start codon and a *Kpn*I site downstream of the stop codon in *AhDGAT2a* (AhD2F-S and AhD2F-A, Supplementary Table S2). *AhDGAT2* was amplified by PCR (30 cycles: 95°C, 30 s; 56°C, 30 s; 1 min 20 s, 72°C) using *Pfu* DNA polymerase. The PCR product was digested with *Bam*HI and *Kpn*I and ligated into the plant binary vector pROK II (Transgen, China). This vector contains a kanamycin resistance gene and a MCS between the *CaMV35S* promoter and the *NOS* terminator between the left and right T-DNA borders. The peanut *DGAT2a* constructs were transformed into competent cells of *Agrobacterium tumefaciens* LBA4404.

Tobacco leaves were cut into approximately 1.0 cm^2^ pieces and soaked in 1/2 × MS liquid medium with an *A. tumefaciens* cell density of about 0.5 (OD_600nm_) for 30 min, then placed on sterile filter paper for several minutes until the liquid medium had dried. Pieces of leaf treated in this manner were transferred to differentiation medium (MS basal medium, 30 g/L glucose, 0.1 mg/L naphthylacetic acid, and 1.0 mg/L 6-benzylaminopurine) supplemented with kanamycin (100 mg/L) as the plant-selective agent, and cefotaxime (250 mg/L) to prevent further bacterial growth. Regenerated shoots were rooted on 1/2 × MS solid medium with sucrose (30 g/L), kanamycin (50 mg/L), and cefotaxime (250 mg/L). Rooted plants were grown in flowerpots under a 16 h light period at 25°C and an 8 h dark period at 18°C. Primary transformants were subcultured as T_0_ plantlets for 6–7 weeks to generate sufficient leaf material for molecular analysis. Positive transformants were transferred to a greenhouse and grown under standard conditions for FA analysis and seed harvest. When the transformants flowered, artificial pollinations were performed daily.

The seeds from the positive transformants were grown on 1/2 × MS medium with 100 mg/L kanamycin to select for positive plants (i.e., the T_1_ generation). Green plants from transformants were kept for seed harvest. T_2_ seeds were transferred to 1/2 × MS medium containing 100 mg/L kanamycin; plants lacking character segregation were chosen to yield seeds. These seeds (T_3_ generation) were continuously selected on 1/2 × MS medium containing 100 mg/L kanamycin; transformants without character segregation were chosen for FA analysis, molecular identification, and expression pattern analysis of FA metabolic genes.

### Molecular Identification of Transgenic Tobacco Plants

To identify the T_3_ transgenic tobacco plants, three pairs of primers (nptII-S and nptII-A, CaMV35S-S and CaMV35S-A, AhD2-FS and AhD2-FA, Supplementary Table S2) were used to amplify the corresponding amplicons from genomic DNA. Plants containing all of these three fragments were considered positive transformants and used as candidates for FA content analysis.

### FA Analysis

Seeds from the transformed tobacco plants and WT were cryodesiccated, accurately weighed, and ground into a powder in a test tube. Each of the yeasts strains were grown in media containing galactose, pelleted by centrifugation and dried. Three technical replicates were conducted for each sample. The methyl ester of heptadecanoic acid (Nu-Chek, United States) was used as a reference standard. Samples and standard were soaked in 2 ml of 2% sulfuric acid in dry methanol for 16 h at room temperature, followed by 80 min of heating at 90°C to convert the FAs into FA methyl esters (FAMEs). After addition of 2 mL of distilled water and 3 mL of hexane, the FAMEs were extracted for analysis by gas chromatography (GC).

The FAME composition of the samples was analyzed using an Agilent Technologies 6890N gas chromatograph (Agilent Company, United States). An initial column temperature of 140°C was maintained for 5 min then increased to a final temperature of 240°C at a rate of 4°C/min, followed by a 10 min hold at 240°C. Injection and detector temperatures were 240 and 26°C, respectively. Two microliters of each sample were injected. FAMEs were identified by comparison of retention times relative to the standards. The results were analyzed using the ChromPerfect^®^ LSi system chromatography software (ChromPerfect^®^ LSi system, United Kingdom) with the FAME mix peak area as the reference.

Fatty acid content was computed as absolute content (mg/g) using the GC area counts for the different FAMEs, based on quantification of known amounts of added internal standards. The quantities of the FAMEs in each sample were used to calculate oil content using the equation:

(1) $$\begin{array}{c} W_{i}=\frac{A_{i}^{*}M_{s}}{A_{s}^{*}M} \end{array}$$

where *M*_s_ is the weight of the internal standard added to a sample, *A*_i_ is the area counts of the individual FAME, *A*_s_ is the area count of the corresponding FAME in the internal standard, and *M* is the weight of the sample used.

### Expression Pattern Analysis of FA Metabolism-Related Genes in Transformed Tobacco Plants

Total RNA was isolated from both WT and transformed tobacco leaves using TRIzol^®^ reagent (Invitrogen, United States) following the manufacturer’s protocol. Five micrograms of total RNA was reverse transcribed using a First-Strand cDNA Synthesis Kit (Fermentas, United States) with an oligo (dT) primer. Q-PCR was performed for 40 cycles of 15 s at 95°C, 1 min at 60°C, and then 15 s at 95°C by ABI7500 Fast Real-Time PCR System machine. *Actin* was used as a loading control. Sequences of the primers used for Q-PCR are provided in Supplementary Table S2.

### Statistical Analysis

All experiments were performed using three replicates. The students’ *t*-test in SPSS was used for determination of significant differences. Values of *p* < 0.05 were considered significant.

## Results

### Cloning Full-Length *AhDGAT2* cDNAs

A pair of primers (Supplementary Table S2) based on the conserved domains of *Medicago truncatula DGAT2* (*MtDGAT2*, BT052205.1) and *Ricinus communis* DGAT2 (*RcDGAT2*, AY916129) nucleotide sequences were designed. A fragment of 197 bp was successfully amplified that exhibited 81.73 and 74.62% identity to the homologous fragments from *MtDGAT2* and *RcDGAT2*, respectively.

Nested primers for 3’RACE were designed to amplify the 3′-end of the target sequence. A 676 bp fragment was amplified; sequencing confirmed that this poly (A) tail fragment comprised the 3′-end of the target sequence. Nested primers for 5′RACE amplified a fragment of about 600 bp, and the sequencing results confirmed that it was the 5′-end of the target gene. Using DNAMAN software, these three fragments (197, 676, and ∼600 bp) aligned into a sequence comprising a 52 bp 5′-UTR, a complete ORF of 1005 bp, and a 175 bp 3′-UTR. We called the full sequence *Arachis hypogaea DGAT2a* (*AhDGDAT2a*).

### Sequence Comparisons of the Different *AhDGAT2* Types

The genetics and botanical classification of the genus *Arachis* is complex: this group is split into nine sections, containing at least 80 species, including wild peanut (*A. hypogaea*) species primarily represented by A-, B-, or K-genome diploid genomes (Krapovickas and Gregory, 1994). Most domesticated peanut species are AABB type allotetraploids. Thus, we were interested to learn if there were any differences in the *DGAT2* nucleotide and AA sequences in the different peanut varieties. Eleven peanut cultivars belonging to five different peanut types were selected for analysis of *AhDGAT2* gene diversity in this experiment (Supplementary Table S1). ‘Luhua 14’ and ‘Luhua 9’ are Runner type peanut cultivars, ‘052106’ and ‘Taishansanlirou’ are Valencia type peanuts, ‘Liguimake’ and ‘Feilongxiang’ are Dragon type peanuts, and ‘Guihua17’ and ‘Taishanzhenzhu’ are Spanish type peanuts. ‘Ad’ is a representative of *A. duranensis*, the A-genome (AA) diploid ancestor of cultivated peanut. ‘A12’ and ‘A026’ are cultivars of *A. glabrata*, a tetraploid of uncertain ploidy type pedigree, due to the presence of various meiotic chromosomal configurations, including as many as 21 different chromosome configurations at diakinesis-metaphase I (Ortiz et al., 2011).

A pair of primers was designed to amplify partial *AhDGAT2* ORFs from all 11 peanut cultivars. Specific primer sequences were designed based on these partial sequences, and full-length cDNA clones of approximately 1173 bp were amplified, cloned into the pMD18-T vector, and sequenced. To ensure the reliability of the results, several cloning and sequencing reactions were performed for each cultivar (Supplementary Table S1). The sequencing results revealed eight different types of *AhDGAT2*, which we have designated *AhDGAT2a-h* (**Supplementary Figure S1** and Table S1, GenBank: JF897614–JF897621, respectively).

**FIGURE 2:**
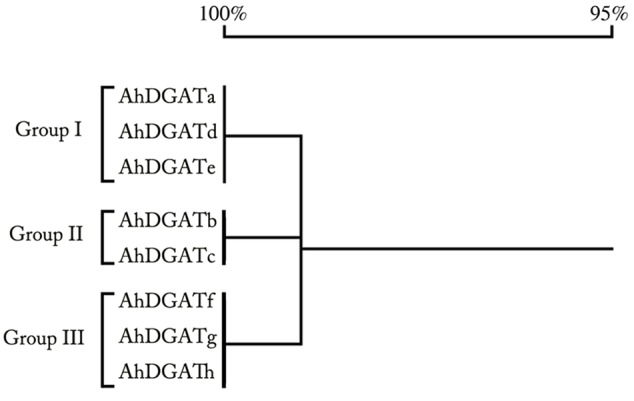
Protein similarity comparison of the eight cloned AhDGAT2 variants.

Inspection of the *AhDGAT2a-h* nucleotide sequence alignments revealed 21 differences in their ORFs (**Supplementary Figure S1**), but only six differences in the deduced AA sequences (**Figure 1**). Using the full-length cDNA sequences (accounting for all 21 of the nucleotide differences) we next performed a phylogenetic comparison that showed the eight AhDGAT2 sequences clustered into three clades (**Figure 2**): *AhDGAT2a, AhDGAT2d*, and *AhDGAT2e* were assigned to group I; *AhDGAT2b* and *AhDGAT2c*, were assigned to group II; whilst *AhDGAT2f, AhDGAT2g* and *AhDGAT2h* made up group III. The numbers and types of specific *AhDGAT2* genes present in each peanut type were not easily predictable. The wild diploid variety ‘Ad’ and *A. glabrata* accession ‘A026’ each contained only a single type of *AhDGAT2* in the results surveyed in Supplementary Table S1, while *A. glabrata* accession ‘A12’ contained representative sequences from four different *AhDGAT2*s. The eight cultivated tetraploid *A. hypogaea* accessions were largely dominated by *AhDGAT2a* and *AhDGAT2b* sequences, in approximately equal proportions, with some exceptions, such as the absence of *AhDGAT2b* in accession ‘Taishanzhenzhu’ and the presence of additional *AhDGAT2* types in ‘Taishansanlirou’ and ‘Feilongxiang’ (Supplementary Table S1).

### Phylogenetic Analysis of Different Plant DGATs

**FIGURE 3:**
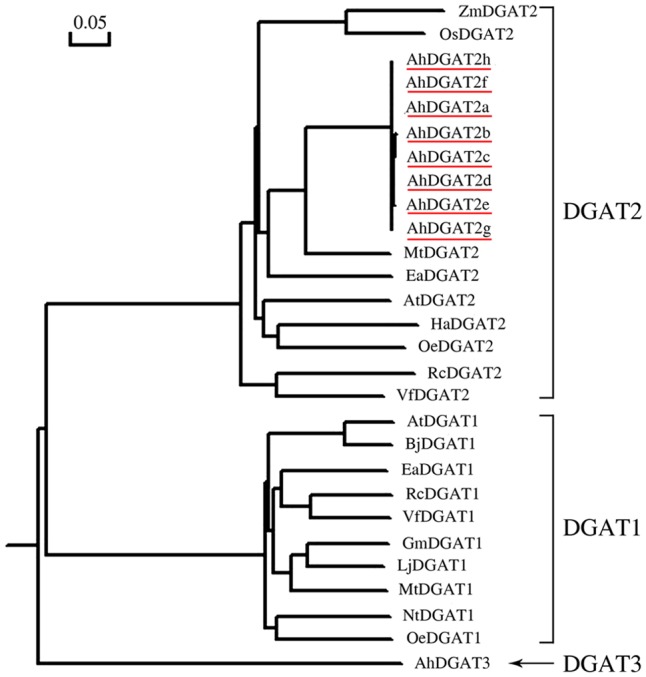
Phylogenetic relationships among the diacylglycerol acyltransferase (DGAT) protein sequences from various plant species. The tree was generated by DNAMAN software. The GenBank protein ID numbers for the listed DGATs are as follows: AhDGAT3, *Arachis hypogaea*, AAX62735.1; AtDGAT1, *Arabidopsis thaliana*, CAB44774.1; AtDGAT2, *A. thaliana*, NP_566952.1; BjDGAT1, *Brassica juncea*, AAY40784.1; EaDGAT1, *Euonymus alatus*, AAV31083.1; EaDGAT2, *E. alatus*, ADF57328.1; GmDGAT1, *Glycine max*, BAE93461.1; HaDGAT2, *Helianthus annuus*, ABU50328.1; LjDGAT1, *Lotus japonicus*, AAW51456.1; MtDGAT1, *Medicago truncatula*, ABN09107.1; MtDGAT2, *M. truncatula*, ACJ84867.1; NtDGAT1, *Nicotiana tabacum*, AAF19345.1; OeDGAT1, *Olea europaea*, AAS01606.1; OeDGAT2, *O. europaea*, ADG22608.1; OsDGAT2, *Oryza sativa* Japonica Group, NP_001057530.1; RcDGAT1, *Ricinus communis*, XP_002514132.1; RcDGAT2, *R. communis*, XP_002528531.1; VfDGAT1, *Vernicia fordii*, ABC94471.1; VfDGAT2, *V*. *fordii*, ABC94473.1; ZmDGAT2, *Zea mays*, NP_001150174.1; AhDGAT2s are underlined.

Phylogenetic comparisons of AhDGAT2a-h protein sequences to other known plant DGATs indicated that the eight AhDGAT2s cluster with other plant DGAT2 sequences, but all peanut sequences share higher sequence identity to each other than to any of the other DGAT2s selected for construction of the tree (**Figure 3**). The closest interspecies relatives are MtDGAT2 and *E. alatus* DGAT2 (EaDGAT2), which share 74.85 and 66.17% AA identity, respectively, to peanut DGAT2.

### Identification of Putative Functional Motifs in AhDGAT2s

The TMHMM program,^1^ predicted two potential transmembrane-spanning helices near the *N*-terminus (**Figure 1**, AAs 40–62 and 67–82), suggesting that their location might be found in the membrane systems of the plant *in vivo*, similar to other characterized plant DGAT2 proteins (Shockey et al., 2006). All eight AhDGAT2s had the same predicted structure that included a small *N*-terminal domain, two transmembrane-spanning domains interrupted by a very small ER lumen-targeted turn region, and a large cytosolic *C*-terminal domain.

The NCBI Conserved Domain Search^2^ confirmed that all AhDGAT2s possess a LPLAT_MGAT-like domain at their *C*-termini (**Figure 1**, AAs 104–321). The presence of the signature putative acyl-acceptor binding pocket domain indicates that AhDGAT2, like many other plant DGAT2s, likely has acyltransferase activity.

### Expression Pattern Analysis of *AhDGAT2* in Developing Seeds and Other Organs

**FIGURE 4:**
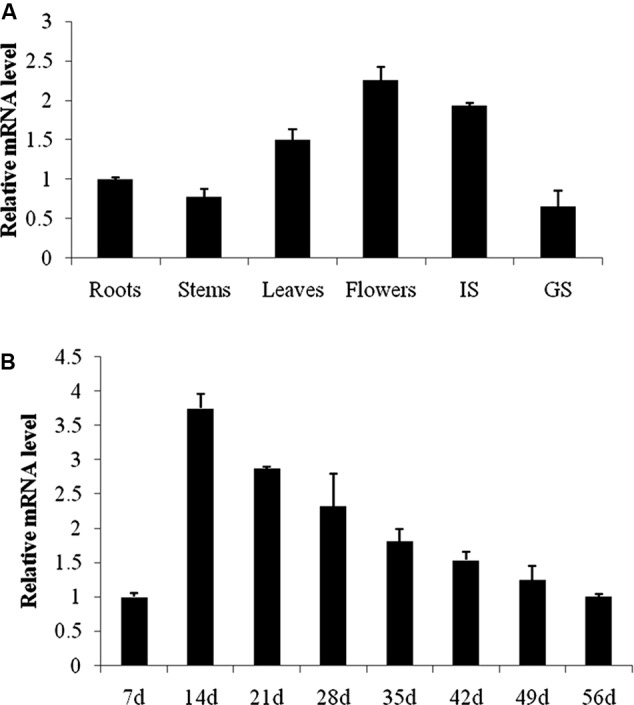
*AhDGAT2* expression pattern analyses. **(A)** Expression patterns of *AhDGAT2* in different peanut organs. IS, immature seeds; GS, germinating seeds. **(B)** Expression patterns of *AhDGAT2* in different developmental stages of peanut seed (The eight stages of peanut seed development corresponding to the intervals after the peanut needles burrow into the soil).

To further investigate the potential role of *AhDGAT2* in TAG biosynthesis, we analyzed the organ-specific and seed stage-specific expression patterns of *AhDGAT2* transcripts in cultivar ‘Luhua14.’ Q-PCR was employed to monitor the expression levels of *AhDGAT2*, using peanut *actin* gene as a reference gene (Chi et al., 2014). Because the *AhDGAT2a* and *AhDGAT2b* nucleotide sequences have only minor differences between them, even in the 5′ and 3′-UTR regions, it was very difficult to design allele-specific primer pairs. Despite several attempts, designing such primers proved unsuccessful. Hence, the transcript levels shown in **Figure 4** represent the sum of both *AhDGAT2a* and *AhDGAT2b* expression. The results of the organ-specific analysis indicated that *AhDGAT2* transcripts were expressed in all of the organs sampled. However, the expression levels were relatively higher in leaves, flowers, and immature seeds, compared to roots, stems, and germinating seeds (**Figure 4A**). *AhDGAT2* was expressed in all seed developmental stages (**Figure 4B**). However, in 14 and 21 days, the *AhDGAT2* expression levels were relatively high. Expression levels gradually declined as the seeds matured.

### Functional Activity Assay of the Six Site-Specific AhDGAT2a Mutants

**FIGURE 5:**
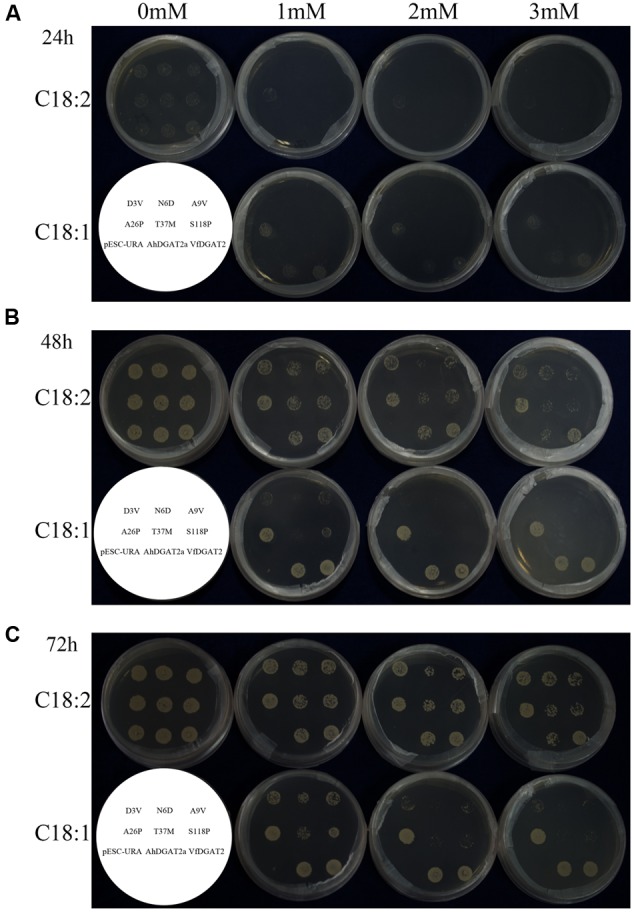
Plate growth lipotoxicity rescue assays. Cells of the TAG- and sterol-deficient quadruple mutant *Saccharomyces cerevisiae* strain H1246 were transformed with plasmids bearing each of the respective plasmids, then cultured in solid media containing oleic or C18:2 at 30°C. **(A)** 24 h; **(B)** 48 h; **(C)** 72 h. The key describing the arrangement of the six AhDGAT2a mutants, the pESC-URA negative control, and the parental AhDGAT2a positive control are shown in the lower left corner of each panel.

To evaluate the ability of the six site-specific AhDGAT2a enzyme mutants to produce TAGs, we used *S. cerevisiae* strain H1246, which lacks all four genes that synthesize TAG, in functional complementation assays (Sandager et al., 2002). Transformed yeast cells were cultivated on solid medium contained C18:1 and C18:2, and relative growth rates observed at different time points. The cell growth status reflects the cells’ ability to detoxify free FAs in the media by metabolizing them into TAG, if the respective strains express an active DGAT enzyme from the transformed plasmid (Siloto et al., 2009). We used empty pESC-URA plasmid as a negative control. All strains, including the negative control, grew well in the absence of FAs. But only the strains expressing the native or mutant AhDGAT2a enzymes grew in the presence of free FAs (**Figure 5**).

Significant differences in cell growth were observed when the variant AAs were introduced into the parental sequence. In media without free FAs, all strains exhibited normal growth (**Figure 5**, 0 mM). Of the peanut DGAT2 strains, only A26P cells had grown on plates supplemented with 1, 2, and 3 mM C18:2 after 24 h (**Figure 5A**, C18:2). On plates supplemented with 1, 2, and 3 mM C18:1, A26P, parental AhDGAT2a, and the VfDGAT1 positive control grew faster than the other five mutants (**Figure 5A**, C18:1). At 48 and 72 h, A26P, AhDGAT2a, and VfDGAT1 still outpaced the other five mutants (**Figures 5B,C**). These results indicate that all six mutants still possess TAG biosynthetic activity, but the enzymatic activity of A26P seems slightly increased.

Another interesting observation is that A26P displayed approximately equal growth on media containing either C18:2 or C18:1, comparable to AhDGAT2a and VfDGAT1. However, the other five mutants have lower apparent activity toward substrates containing C18:1 than C18:2 (**Figures 5B,C**), given the higher growth rates on equal concentrations of l C18:2 versus C18:1. These results suggest that single AA substitutions may affect the substrate preference of peanut DGAT2 enzymes.

### Nile Red Assay of Transgenic Yeast Cells

**FIGURE 6:**
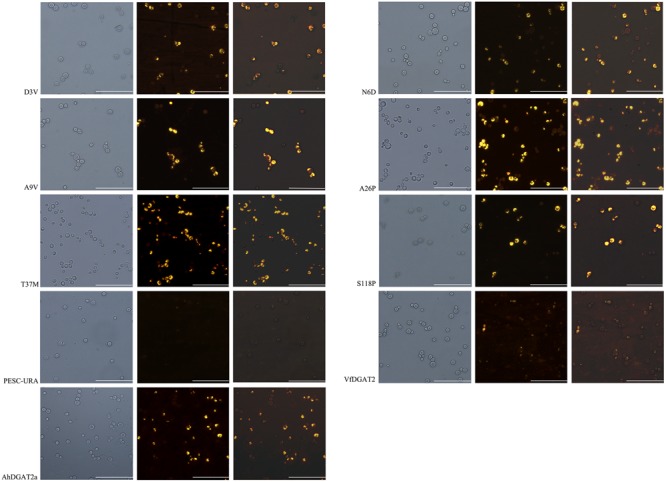
Nile Red staining and fluorescence images of the transformed yeast strains. Bar = 50 μm. The left column was bright-field images, the middle was images of Nile Red fluorescence images, and the right panels were merged with the bright-field and fluorescence images.

Complex lipid biosynthetic capacity of the six AhDGAT2a mutants was also assessed by visualization of lipid droplets in transgenic yeast using the lipophilic fluorescent dye Nile Red (Siloto et al., 2009). The AhDGAT2 lines were compared to the empty pESC-URA plasmid negative control strain, and the VfDGAT1 positive control (Shockey et al., 2006). Lipid droplets were not observed in the negative control (**Figure 6**). The parental AhDGAT2a and all six single residue variants produced lipid droplets similar to the VfDGAT1 positive control (**Figure 6**). Qualitative visual inspection suggested that all transgenic AhDGAT2 strains showed similar fluorescence intensities indicating approximately equal lipid production. But the A26P variant seemed to show higher fluorescence intensity than the parental DGAT2a and other variants (**Figure 6**). To verify these observations, Nile Red fluorescence was also quantified spectrophotometrically. These analyses confirmed that all transgenic yeast strains had significantly higher fluorescence intensity than the negative control (*P* < 0.05 for T37M, *P* < 0.01 for all others) (**Supplementary Figure S2**). The A26P and N6D mutants also had higher fluorescence intensities than the parental AhDGAT2a strain (at *P* < 0.05 and *P* < 0.01, respectively) (**Supplementary Figure S2**).

### FA Analysis of the Transgenic Yeast Cells

**FIGURE 7:**
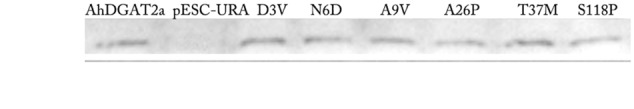
Western blot of the transformed yeast strains. Yeast actin is used a load control.

**FIGURE 8:**
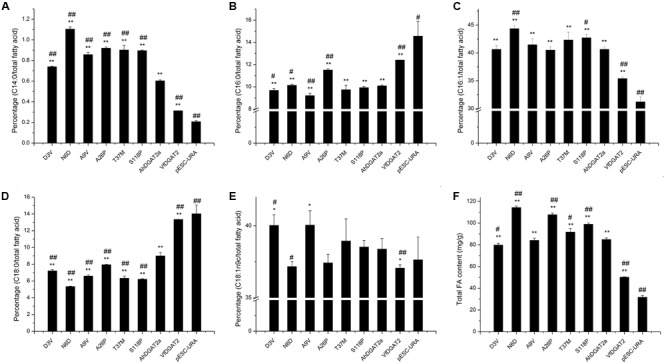
Gas chromatography quantification of total lipids and individual fatty acids (FAs) in *AhDGAT2* transgenic yeast strains. The values shown are the averages of 3–4 measurements from each yeast strain; error bars represent the standard error of measurement. ^∗^Compared to pESC-URA, student’s unpaired *t*-test, *P* < 0.05; ^∗∗^Compared to pESC-URA, *P* < 0.01; ^#^Compared to AhDGAT2a, *P* < 0.05; ^##^Compared to AhDGAT2a, *P* < 0.01. **(A)** C14:0; **(B)** C16:0; **(C)** C16:1; **(D)** C18:0; **(E)** C18:1; **(F)** total lipids.

The transgenic protein expression levels of the parental AhDGAT2a and six mutants were analyzed via western blotting. All constructs tested expressed at approximately equal levels (**Figure 7**). All transgenic yeast strains contained elevated total FA content compared with the negative control strain (**Figure 8F**). The total FA content of the D3V mutant decreased slightly, whereas that of the N6D, A26P, T37M, and S118P strains significantly increased (∼14–26%), relative to the AhDGAT2a parental strain. These results agreed with the results of plate growth and Nile Red staining assays (**Figure 6** and **Supplementary Figure S2**).

The six mutants of *AhDGAT2a* not only significantly changed the total FA content, but also had some effect on FA composition (**Figures 8A–E**). Four FAs accounted for 97.0–98.2% of the total FA content. C16:1 was the most abundant, comprising 40.5–44.3% of total FAs in transgenic AhDGAT2 yeasts; followed by C18:1n9 (37.1–40%), C16:0 (palmitic acid, 9.2–11.5%), and C18:0 (stearic acid, 5.3–9.0%) (**Figure 8**). D3V and A9V significantly decreased the content of C16:0 compared to AhDGAT2a yeast, but A26P significantly increased its content. N6D and S118P significantly increased the content of C16:1 compared to AhDGAT2a. C18:0 content was decreased significantly in all mutants compared to AhDGAT2a. C18:1n9 content increased in D3V, but decreased in the N6D mutant (**Figure 8**).

### Analysis of WT and Transformed Tobacco Leaf and Seed FAs

**FIGURE 9:**
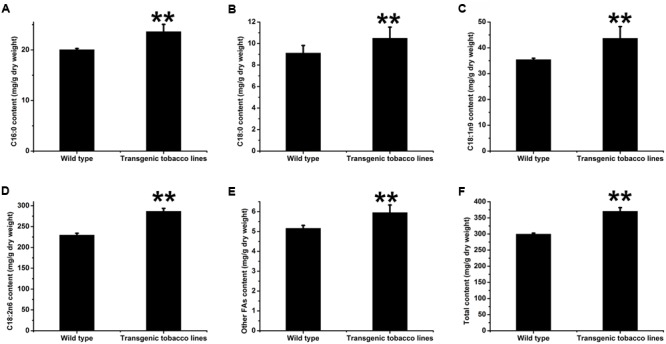
Total FA content of wild-type (WT) and *AhDGAT2a*-transgenic tobacco seeds. Total lipids from seed samples representing three technical replicates from each of three WT and nine homozygous T_3_ transgenic lines were converted to FA methyl esters (FAMEs) and quantified by gas chromatography (GC), as described in the text. ^∗^Compared to WT, student’s unpaired *t*-test, *P* < 0.05; ^∗∗^*P* < 0.01. **(A)** C16:0 content comparison; **(B)** C18:0 content comparison; **(C)** C18:1n9 content comparison; **(D)** C18:2n6 content comparison; **(E)** Other FAs content comparison; **(F)** Total FAs content comparison.

We were also curious to study the effects of over-expression of AhDGAT2 on lipid metabolism *in planta*. Wild-type tobacco was transformed with *AhDGAT2a*, expressed behind the *CaMV35S* promoter. We obtained nine distinct homozygous T_3_ transformants with a single-copy transgene insertion. T_3_ plants from all nine events were grown side-by-side with plants from three WT parents, leaves, and mature seeds were harvested from all 12 lines and analyzed for changes in FA content by GC (**Figure 9**). Twenty different FAs were detected in the WT tobacco seeds; among them, four FAs accounted for 98.4–98.7% of the total FA content. C18:2n6 was the most abundant, comprising 76.2–79.4% of total FAs, followed by C18:1n9 (10.2–13.1%), C16:0 (5.8–7.2%), and C18:0 (2.5–3.0%). The other FAs include C16:1, C18:3n3, C20:0, C20:1, C22:0, and C24:0, each of which is present in very small amounts in these samples. *AhDGAT2a* overexpression significantly increased tobacco seed total FA content (**Figure 9F**), including increases in all four of the predominant FAs (and the sum of the other trace FAs as well, **Figures 9A–E**). The total FA content was increased by ∼20% on average, with 10–45% relative increases in each of the four major FAs.

**FIGURE 10:**
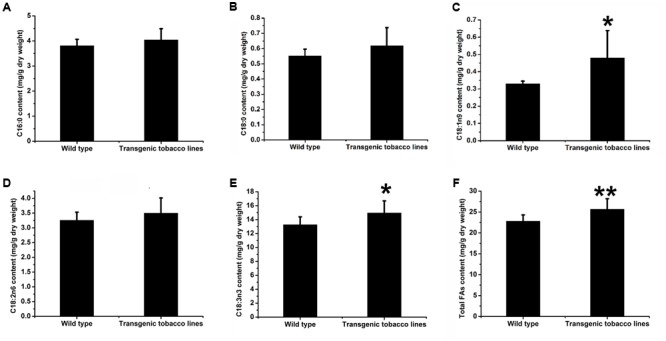
Total FA content of WT and *AhDGAT2a*-transgenic tobacco leaves. Total lipids from fresh, fully expanded leaf samples representing three technical replicates from each of three WT and nine homozygous T_3_ transgenic lines were converted to FAMEs and quantified by GC, as described in the text. ^∗^Compared to WT, student’s unpaired *t*-test, *P* < 0.05; ^∗∗^*P* < 0.01. **(A)** C16:0 content comparison; **(B)** C18:0 content comparison; **(C)** C18:1n9 content comparison; **(D)** C18:2n6 content comparison; **(E)** C18:3n3 content comparison; **(F)** Total FAs content comparison.

Given that the *CaMV35S* promoter fused to the AhDGAT2a transgene is typically expressed even more strongly in plant vegetative tissues relative to seeds, we also explored the FA composition and content of WT and transgenic leaves. Total leaf FA levels were increased by 12.56% in *AhDGAT2a* overexpressors (**Figure 10F**) due to slight increases in C16:0, C18:0, and C18:2n6 (**Figures 10A,B,D**), and significant increases in C18:1n9 and C18:3n3 (**Figures 10C,E**).

### Expression Patterns of 10 FA-Related Genes in WT and Transgenic Tobacco Plants

**FIGURE 11:**
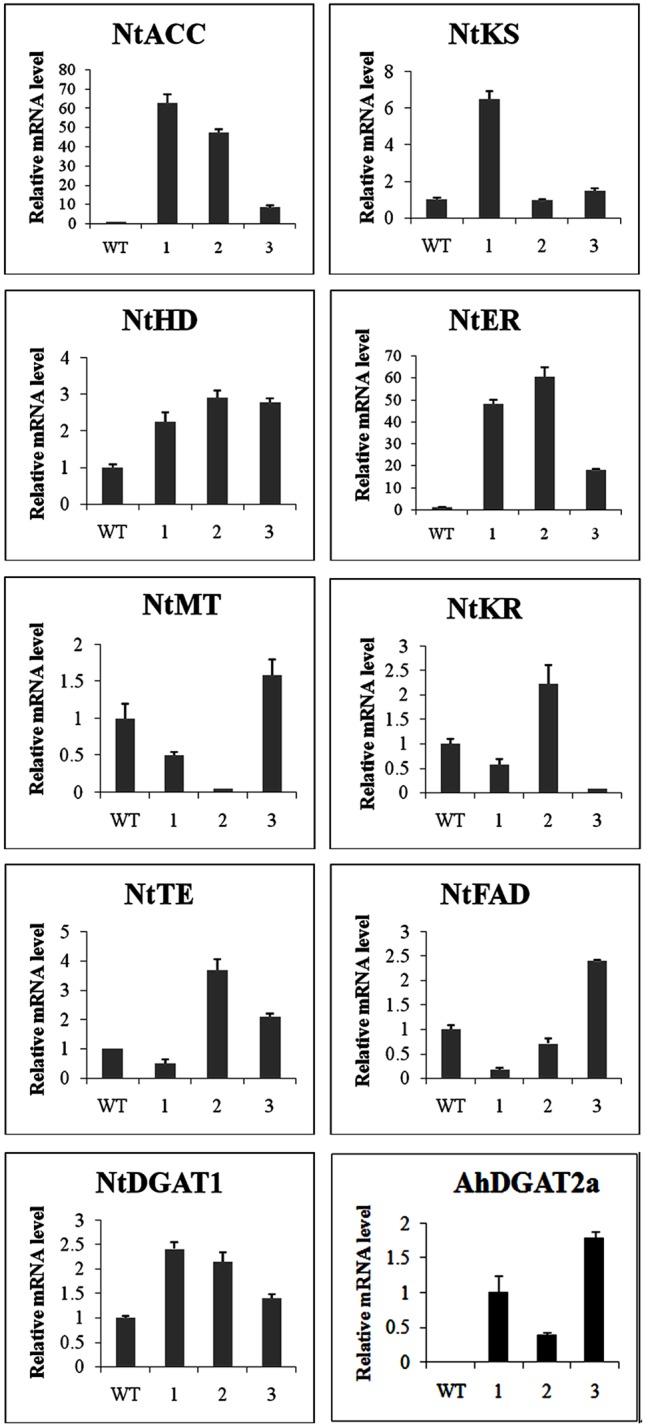
Analysis of gene expression patterns of nine endogenous FA metabolism-related genes and transgenic *AhDGAT2a*, in leaves of different transgenic tobacco lines, as measured by Q-PCR. WT, wild-type tobacco; 1, 2, and 3 represent three transgenic tobacco lines, chosen at random from the population of lines used in **Figures 9** and **10**. Transcript levels were quantified by comparison to that of a peanut *actin* reference gene (Chi et al., 2014). The identity of each gene is described in the “Results.”

Overexpression of *AhDGAT2a* in transgenic tobacco lines caused obvious changes in total lipid synthesis, as shown in **Figures 9** and **10**. To investigate possible underlying alterations in the genetic control of lipid metabolism in these lines, transcript levels for several endogenous genes were quantified in leaves of three randomly chosen transgenic lines. Lipid metabolism is a highly complicated process, involving many genes (**Supplementary Figure S3**). We chose nine tobacco FA- and storage lipid metabolism-related genes (representing early, middle, and late stages of FA flux toward TAG) which were annotated in NCBI, namely acetyl-CoA carboxylase (*NtACC*, JQ267734), β-keto-ACP synthase (*NtKS*, JQ267736), malonyl-CoA-ACP transferase (*NtMT*, JQ267740), β-ketoacyl-ACP reductase (*NtKR*, JQ267735), β-hydroacyl-ACP dehydratase (*NtHD*, JQ267738), enoyl-ACP reductase (*NtER*, JQ267739), thioesterase (*NtTE*, JQ267737), omega-6-desaturase (*NtFAD*, AY660024), and type-1 diacylglycerol acyltransferase (*NtDGAT1*, AF129003). Their transcript levels were analyzed by Q-PCR (**Figure 11**). The presence of *AhDGAT2* transcripts in the leaves of over-expressing lines (and the absence of the transgene in untransformed controls) was also tested. The primer sequences used are shown in Supplementary Table S2. Transcripts from each of these genes were detected in all of the transgenic lines examined, but the expression patterns slightly differed from each other in some cases.

Compared to WT tobacco, *NtACC, NtHD, NtDGAT1*, and *NtER* expression increased in the leaves of all three transgenic lines; while *NtKS, NtMT, NtKR, NtTE*, and *NtFAD* transcript levels were higher in one or two of the three transgenic lines analyzed (**Figure 10**).

*AhDGAT2a*-specific primers did not amplify any related endogenous sequences in WT tobacco. These results suggest that heterologous expression of *AhDGAT2a* in tobacco plants could positively influence the expression pattern of multiple FA-related genes, thus enhancing the FA content of transgenic lines.

## Discussion

Peanut germplasm is abundant and highly diversified in China, and many peanut varieties were cultivated by radiation breeding, which increased the genetic diversity. There are many differences among peanut types with regard to traits such as oil content, seed color, size, and shape, as well as plant morphology. However, previous studies have come to different conclusions regarding the degree of variability at the level of genes, transcript levels, and single nucleotide polymorphisms in wild diploids compared to cultivated allotetraploid peanut species (Chopra et al., 2016). The survey of *AhDGAT2* sequences summarized in Supplementary Table S1 and Figure S1 suggests that both cultivated and wild peanut species collectively contain multiple isoforms of AhDGAT2, but all share highly conserved primary sequences and thus would likely contribute very little to any observed genetic variability.

As discussed in the “Introduction,” it is clear that the relative roles of DGAT1 and DGAT2 are complex, and may vary considerably between different plant species. Chi et al. (2014) identified three novel *AhDGATs* genes from peanut. The expression level of *AhDGAT1-1* and *AhDGAT3-3* were higher in flowers than in the other tissues examined, whereas the *AhDGAT1-2* transcript was more abundant in roots, seeds, and cotyledons (Chi et al., 2014). The complexities of the interwoven pathways of lipid synthesis are complicated further by the recent discovery of a third family of DGAT enzymes, called DGAT3 (Saha et al., 2006). The precise biological roles of plant DGAT3s are even less certain, but available evidence suggests it may function as part of a cytosolic pathway that balances membrane lipid and TAG biosynthesis by regulating acyl-CoA pool size and composition (Hernández et al., 2012). As such, plant DGAT3s are often expressed in vegetative tissues, whereas other DGATs enzymes with known roles in TAG biosynthesis are often expressed preferentially in developing seeds [>15x higher expression in seeds versus leaves for tung and castor DGAT2 (Kroon et al., 2006; Shockey et al., 2006)]. *AhDGAT2* expression was distributed across all organs analyzed (**Figure 4**). These results suggest that *AhDGAT2* has evolved to fill both ‘seed-specific’ and general housekeeping roles. The totality of existing evidence strongly suggests that AhDGAT1, 2 and 3 all contribute to peanut lipid metabolism, but the functional division of each AhDGAT type requires further study.

In a previous study, we over-expressed AhDGAT2a and AhDGAT2b in *Escherichia coli* and showed that both enzyme isoforms increased the FA content (Peng et al., 2013) of transformed bacteria. Here, we over-expressed a representative gene (*AhDGAT2a*) in transgenic yeast. AhDGAT2a activity rescued FA-induced growth arrest in the TAG-deficient yeast strain H1246, when either C18:1 or C18:2 was used in the media formulations (**Figure 5**); the exogenous substrates were metabolized into neutral lipids and packaged into lipid droplets in all active DGAT strains (**Figure 6**).

We also showed that over-expression of *AhDGAT2a* in transgenic tobacco seeds and leaves caused significant increases in total FA content on a dry weight basis (**Figures 9, 10**). The types of lipids that accumulated in transgenic tobacco leaves and seeds was not determined, but it is very likely that the majority was packaged in the form of neutral lipid droplets (or oil bodies, as they are typically referred to in plants) containing DAGs and TAGs, which is supported by evidence of elevated levels of expression for numerous genes in the early, middle, and later stages of FA and complex lipid biosynthesis (**Figure 11**). Increased gene expression was especially pronounced for acetyl-CoA carboxylase (NtACC) and enoyl-ACP reductase (NtER) (**Figure 11**), which encode the rate-limiting step and another important early step in FA biosynthesis, respectively (Slabas and Fawcett, 1992; O’Hara et al., 2007). Overall, our results were similar to those recently described by Chen et al. (2015), who overexpressed peanut LPAT2, another important Kennedy pathway enzyme, in transgenic Arabidopsis.

We consider the findings in **Figure 11** particularly interesting. The upregulation of genes in the FA biosynthetic pathway suggests that the substantial increases in FA content shown in **Figures 9** and **10** could represent a redirection of flux of existing fixed carbon. Given the increases in FA content, *AhDGAT2a* overexpression likely also results in increased overall FA synthesis and total lipid carbon flux, thus providing more substrate for neutral lipid biosynthesis than is present in WT seeds. Transcriptional, or post-transcriptional, upregulation of endogenous tobacco *DGAT*s could possibly account for some of the observed changes in FA content as well. In plants, along with seeds, green biomass is as an attractive platform for the production of oils and other specialty lipids, and many recent studies have demonstrated the utility of plant DGAT enzymes to increase leaf, root, and stem oil content (Sanjaya et al., 2011; Vanhercke et al., 2013, 2014). Our results suggest that *AhDGAT2* genes could be valuable components of other lipid biomass engineering strategies in the future.

In the current study, we identified eight closely related *AhDGAT2* gene variants from different peanut cultivars, with only six AA changes among them. Five of the predicted differences are present near the extreme *N*-terminus, upstream of the first transmembrane-spanning domain. This region varies considerably among plant DGAT2s generally (Shockey et al., 2006). Only the S118P variant occurred in the large (and still largely undefined) LPLAT_MGAT-like domain (**Figure 1**). Four of the six mutant enzymes (N6D, A26P, T37M, and S118P) increased the total cellular FA level of transgenic yeast (**Figure 8**). Through the plate growth assay, Nile Red staining, and FA quantification analyses, we found compelling evidence that the N6D and A26P substitutions significantly increased the enzymatic activity level, relative to parental AhDGAT2a (**Figures 4–6**). Future studies will focus on determining whether these two residue changes affect enzyme activity by altering substrate specificity, enzyme stability, or other factors. We will also begin to stack multiple AA substitutions together to assess whether the changes in enzyme properties seen here are additive.

In summary, we investigated genetic diversity of the *AhDGAT2* gene family from eleven different cultivars of peanut, including *A. hypogaea, A. duranensis*, and *A. glabrata*, and discovered eight sequence variants. There are a total of six AA differences in the predicted AA sequences; alteration of parental AhDGAT2a to match each of the single residue variants changed the enzymatic activity in some cases, especially for N6D and A26P. *AhDGAT2* is expressed in all peanut plant organs analyzed, with highest transcript levels in leaves, flowers, and immature seeds. *AhDGAT2a* overexpression in WT tobacco substantially increased seed FA content and altered transcript levels of various endogenous lipid metabolic genes. Overall, these results indicate that the *AhDGAT2* gene family has evolved unique properties compared to other known plant *DGAT*s, and represents a diverse resource that could be useful in determination of new insights to DGAT2 structure, biochemistry, physiological roles, and biotechnological applications.

## Author Contributions

Conceived and designed the experiment: LZ, ZP, GC, and SW. Performed the experiment: LZ, ZP, FB, and LS. Analyzed data: LZ, GC, LS, XL, and SW. Wrote the paper: LZ, JS, FB, XL, and SW. All authors revised the draft and approved the final manuscript.

## Conflict of Interest Statement

The authors declare that the research was conducted in the absence of any commercial or financial relationships that could be construed as a potential conflict of interest.

## Footnotes

1 http://www.cbs.dtu.dk/services/TMHMM/

2 http://www.ncbi.nlm.nih.gov/Structure/cdd/wrpsb.cgi

## Supplementary Material

FIGURE S1 Nucleotide sequence comparison of AhDGAT2a-h.

FIGURE S2 Quantification of Nile Red fluorescence intensity of yeast strains expressing variants of peanut DGAT2a. The values shown are the averages of four measurements from each yeast strain; error bars represent the standard error of measurement. ^∗^Compared to pESC-URA, student’s unpaired *t*-test, *P* < 0.05; ^∗∗^Compared to pESC-URA, *P* < 0.01; ^#^Compared to AhDGAT2a, *P* < 0.05; ^##^Compared to AhDGAT2a, *P* < 0.01.

FIGURE S3 A diagram of the FA metabolism in plant. (1) acetyl-CoA carboxylase (ACC); (2) malonyl-CoA:ACP malonyltransferase (MT); (3) ketoacyl-ACP (KSIII synthase); (4) ketoacyl-ACP synthase (KSI); (5) ketoacyl-ACP reductase (KR); (6) hydroxyacyl-ACP dehydrase (HAD); (7) enoyl-ACP reductase (ER); (8) ketoacyl-ACP synthase (KSII); (9) stearoyl-ACP desaturase (SAD); (10) fatty acyl thioesterase (TE); (11) glycerol-3-phosphate acyltransferase (GPAT); (12) 2 lysophosphatidic acid acyltransferase (LPAAT); (13) phosphatidate phosphatase (PP); (14) diacylglycerol cholinephosphotransferase (DAG-CPT); (15) 2-lysophosphatidylcholine acyltransferase (LPCAT); (16) oleate desaturase (FAD2); (17) linoleate desaturase (FAD3); (18) phospholipid:diacylglycerol acyltransferase (PDAT); (19) acyl-CoA:diacylglycerol acyltransferase (DGAT); (20) diacylglycerol transacylase (DAGTA).
