# Supplementary figures and images for: Variant Amino Acid Residues Alter the Enzyme Activity of Peanut Type 2 Diacylglycerol Acyltransferases
Source: Front Plant Sci. 2017 Oct 16;8:1751. doi: 10.3389/fpls.2017.01751 (PMC5650624; doi:10.3389/fpls.2017.01751)

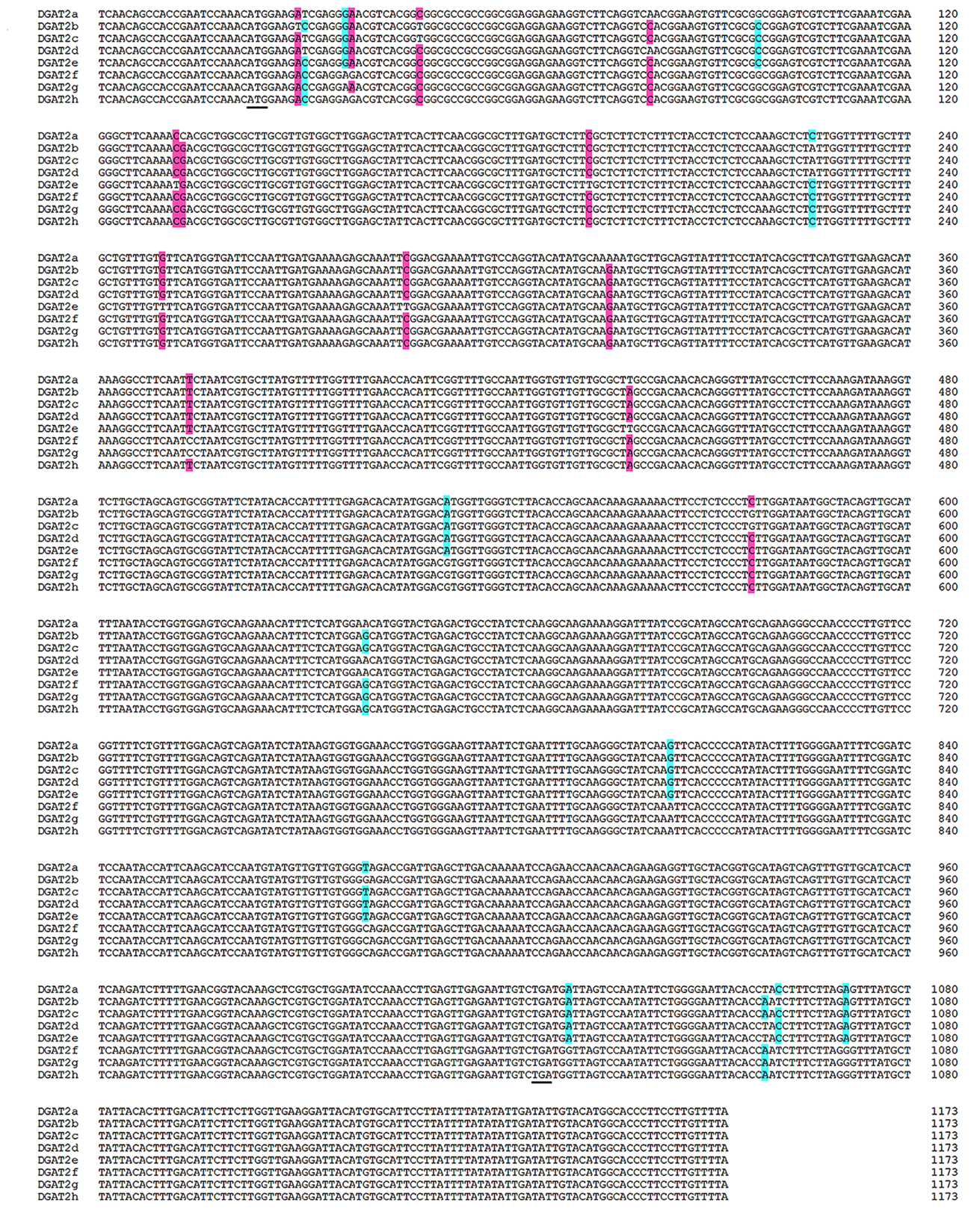

Supplement: FIGURE S1 — Nucleotide sequence comparison of AhDGAT2a-h. [file Image_1.TIF]

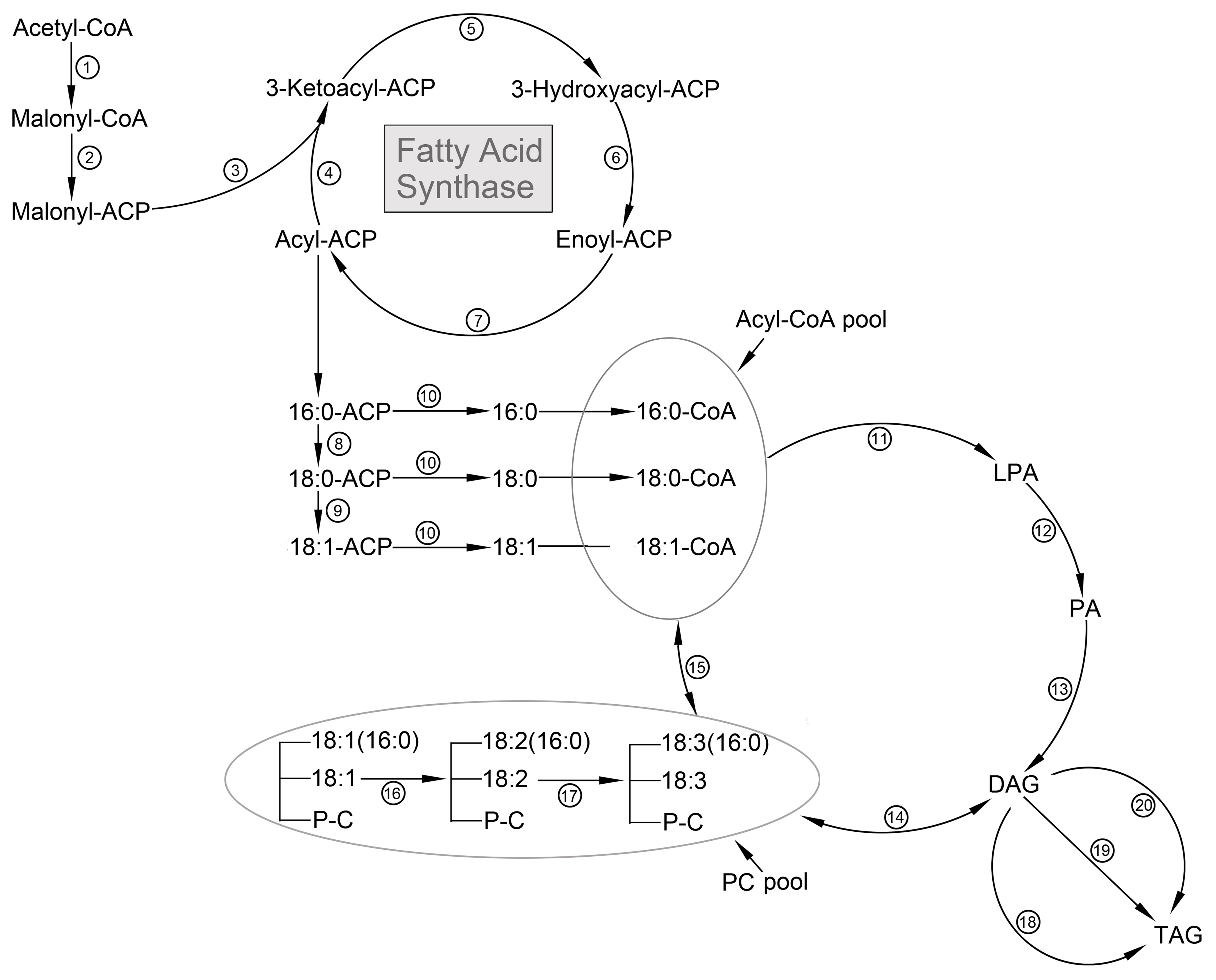

Supplement: FIGURE S3 — A diagram of the FA metabolism in plant. (1) acetyl-CoA carboxylase (ACC); (2) malonyl-CoA:ACP malonyltransferase (MT); (3) ketoacyl-ACP (KSIII synthase); (4) ketoacyl-ACP synthase (KSI); (5) ketoacyl-ACP reductase (KR); (6) hydroxyacyl-ACP dehydrase (HAD); (7) enoyl-ACP reductase (ER); (8) ketoacyl-ACP synthase (KSII); (9) stearoyl-ACP desaturase (SAD); (10) fatty acyl thioesterase (TE); (11) glycerol-3-phosphate acyltransferase (GPAT); (12) 2 lysophosphatidic acid acyltransferase (LPAAT); (13) phosphatidate phosphatase (PP); (14) diacylglycerol cholinephosphotransferase (DAG-CPT); (15) 2-lysophosphatidylcholine acyltransferase (LPCAT); (16) oleate desaturase (FAD2); (17) linoleate desaturase (FAD3); (18) phospholipid:diacylglycerol acyltransferase (PDAT); (19) acyl-CoA:diacylglycerol acyltransferase (DGAT); (20) diacylglycerol transacylase (DAGTA). [file Image_3.TIF]
