# Supplementary material for: Variant Amino Acid Residues Alter the Enzyme Activity of Peanut Type 2 Diacylglycerol Acyltransferases
Source: Front Plant Sci. 2017 Oct 16;8:1751. doi: 10.3389/fpls.2017.01751 (PMC5650624; doi:10.3389/fpls.2017.01751)
Supplement: Supplementary file 4 [file Table_1.DOCX]

**Table S1.** Peanut cultivars investigated in this study.

| Cultivar | Species | Sequencing number^#^ | *AhDGAT2* types and ratios* | Peanut cultivar | Ploidy |
| --- | --- | --- | --- | --- | --- |
| Luhua14 | *A. hypogaea* | 20 | a:b=10:10 | Runner type | [allotetraploid](javascript:void(0);) (AABB) |
| Luhua9 | *A. hypogaea* | 10 | a:b=9:1 | Runner type | [allotetraploid](javascript:void(0);) (AABB) |
| 052106 | *A. hypogaea* | 17 | a:b=7:10 | Valencia type | [allotetraploid](javascript:void(0);) (AABB) |
| Taishansanlirou | *A. hypogaea* | 16 | a:b:c=8:6:2 | Valencia type | [allotetraploid](javascript:void(0);) (AABB) |
| Liguimake | *A. hypogaea* | 12 | a:b=7:5 | Dragon type | [allotetraploid](javascript:void(0);) (AABB) |
| Feilongxiang | *A. hypogaea* | 24 | a:b:c:d=8:11:2:3 | Dragon type | [allotetraploid](javascript:void(0);) (AABB) |
| Guihua17 | *A. hypogaea* | 10 | a:b=5:5 | Spanish type | [allotetraploid](javascript:void(0);) (AABB) |
| Taishanzhenzhu | *A. hypogaea* | 10 | a=10 | Spanish type | [allotetraploid](javascript:void(0);) (AABB) |
| Ad | *A. duranensis* | 9 | a=9 | Wild type | Diploid (AA) |
| A12 | *A. glabrata*** | 23 | a:f:g:h=10:10:1:2 | Wild type | [autotetraploid](javascript:void(0);) (AAAA) |
| A026 | *A. glabrata*** | 7 | e=7 | Wild type | [autotetraploid](javascript:void(0);) (AAAA) |

Note: # The number of individual clones that have been sequenced. *Gene types refer to AhDGAT2a - h classification as designated in Figure S1. Ratios refer to isolate numbers of each gene type present in specific cultivars. **: *A. glabrata* is thought to be an autopolyploid with different degrees of diploidization ([Ortiz et al., 2011](#_ENREF_34)).
