# Supplementary material for: Variant Amino Acid Residues Alter the Enzyme Activity of Peanut Type 2 Diacylglycerol Acyltransferases
Source: Front Plant Sci. 2017 Oct 16;8:1751. doi: 10.3389/fpls.2017.01751 (PMC5650624; doi:10.3389/fpls.2017.01751)
Supplement: Supplementary file 5 [file Table_2.DOCX]

**Table S2.** Primers used in this study

| Primer name | Primer sequence (5’→3’) | Target sequence |
| --- | --- | --- |
| AhD2-S | TCTTACACCAGCAACAAGGAAA | *AhDGAT2* central fragment |
| AhD2-A | GACCAAAGCAGAAAACAGGAAC |  |
| AhD2-3O | TCTTACACCAGCAACAAGGAAA | *AhDGAT2* 3’RACE |
| AhD2-3I | CCCTCTTGGATAATGGCTACAGTTG |  |
| AhD2-5O | ACTGTAGCCATTATCCAAGAGGG | *AhDGAT2* 5’RACE |
| AhD2-5I | TTTCTTTGTTGCTGGTGTAA |  |
| AhD2-FS | TCAACAGCCACCGAATCCA | *AhDGAT2* ORF, Molecular identification of the transformants |
| AhD2-FA | TAAAACAAGGAAGGGTGCCA |  |
| AhD2-SF | AGCCACCGAATCCAAACA | *AhDGAT2* Q-PCR |
| AhD2-SR | CGCCGTTGAAGTGAATAGC |  |
| AhActin-S | AAAGATGGCAGAAGCCGA | Peanut actin for Q-PCR |
| AhActin-A | ACAATACTGGGAAACACAGCAC |  |
| AhD2F-S | CGCGGATCCTCAACAGCCACCGAATCCA | Construction of plasmids for overexpression |
| AhD2F-A | CGGGGTACCTAAAACAAGGAAGGGTGCC |  |
| nptII-S | ATACCGTAAAGCACGAGGAAG | Molecular identification of the transformants |
| nptII-A | TGACTGGGCACAACAGACAAT |  |
| CaMV35S-S | GCTCCTACAAATGCCATCA |  |
| CaMV35S-A | GATAGTGGGATTGTGCGTCA |  |
| DGAT2AF | CGCCGAGATCTACATGGAAGATCGAGGGAACG | Site-specific mutant of *AhDGAT2a* |
| DGAT2A-1F | CGCCGAGATCTACATGGAAGTCCGAGGGAA | Site-specific mutant, D3V |
| DGAT2A-3F | CGCCGAGATCTACATGGAAGATCGAGGGAACGTCACGGTGG | Site-specific mutant, A9V |
| DGAT2AR | CCGCCTTAATTAATCATCAGACAATTCTCAACTT | Site-specific mutant of *DGAT2a* |
| NtACCF | TTGTGGCAAATCTGCTCTTC | Tobacco NtACC for Q-PCR |
| NtACCR | CGAATCACACGAACAGCAAT |  |
| NtKSF | CACAACTCAAATACTTGGTGGG | Tobacco NtKS for Q-PCR |
| NtKSR | ACACTCAACCCTTTGTATGCCT |  |
| NtMTF | TCAACACCATCCACTTAGTCG | Tobacco NtMT for Q-PCR |
| NtMTR | GGAGAGAAATGGAAGGAAGAAG |  |
| NtKRF | CACTTTACTCCGTTCAATGGC | Tobacco NtKR for Q-PCR |
| NtKRR | AAGACGGAGATGCGTAAGC |  |
| NtHDF | TCAATGGCTTCTATGGCTGT | Tobacco NtHD for Q-PCR |
| NtHDR | TTGAGCATTGAACTGGGC |  |
| NtERF | CGTAGCGGCAAAGAACTAATC | Tobacco NtER for Q-PCR |
| NtERR | GCAGGGTGAGTCGTTGTCTA |  |
| NtTEF | ATGGTGGTCACTGCTGCTAC | Tobacco NtTE for Q-PCR |
| NtTER | AGCAGGCTTCTTAGCATTGAG |  |
| NtFADF | AAAGGTTTGGGATTGACGAG | Tobacco NtFAD for Q-PCR |
| NtFADR | GAGAGACCGCTGAAAGCAGTGA |  |
| NtDGAT1F | CGGCGAAAGTAAGCAATG | Tobacco NtDGAT1 for Q-PCR |
| NtDGAT1R | CCTCCGAACCGAGTGATTA |  |
| NtActinF | TTCAGCCACTCGTCTGTGA | Tobacco NtActin for Q-PCR |
| NtActinR | CACACCAAGAAGTCACGAGTTC |  |
